# Neonatal overfeeding disrupts pituitary ghrelin signalling in female rats long-term; Implications for the stress response

**DOI:** 10.1371/journal.pone.0173498

**Published:** 2017-03-10

**Authors:** Luba Sominsky, Ilvana Ziko, Sarah J. Spencer

**Affiliations:** School of Health and Biomedical Sciences RMIT University, Melbourne, Victoria, Australia; Monash University, AUSTRALIA

## Abstract

The hypothalamic-pituitary-adrenal (HPA) axis responses to psychological stress are exacerbated in adult female but not male rats made obese due to overfeeding in early life. Ghrelin, traditionally known for its role in energy homeostasis, has been recently recognised for its role in coordinating the HPA responses to stress, particularly by acting directly at the anterior pituitary where the growth hormone secretagogue receptor (GHSR), the receptor for acyl ghrelin, is abundantly expressed. We therefore hypothesised that neonatal overfeeding in female rats would compromise pituitary responsiveness to ghrelin, contributing to a hyperactive central stress responsiveness. Unlike in males where hypothalamic ghrelin signalling is compromised by neonatal overfeeding, there was no effect of early life diet on circulating ghrelin or hypothalamic ghrelin signalling in females, indicating hypothalamic feeding and metabolic ghrelin circuitry remains intact. However, neonatal overfeeding did lead to long-term alterations in the pituitary ghrelin system. The neonatally overfed females had increased neonatal and reduced adult expression of GHSR and ghrelin-O-acyl transferase (GOAT) in the pituitary as well as reduced pituitary responsiveness to exogenous acyl ghrelin-induced adrenocorticotropic hormone (ACTH) release *in vitro*. These data suggest that neonatal overfeeding dysregulates pituitary ghrelin signalling long-term in females, potentially accounting for the hyper-responsive HPA axis in these animals. These findings have implications for how females may respond to stress throughout life, suggesting the way ghrelin modifies the stress response at the level of the pituitary may be less efficient in the neonatally overfed.

## Introduction

Childhood obesity affects more than 40 million people worldwide and is a significant risk factor for adult obesity and myriad co-morbid diseases and disorders, including hypothalamic-pituitary-adrenal (HPA) axis dysfunction [1]. We have previously shown that neonatal overfeeding in a rodent model induces an overweight phenotype that is maintained into adulthood in both males and females [2–4], with similar increases in fat mass and circulating leptin between the sexes [2, 4, 5]. Both male and female rats overfed *in utero* or postnatally show similarly exacerbated febrile and pro-inflammatory cytokine responses to an immune challenge compared with controls [2, 6], and similar changes in hypothalamic microglial profiles during the neonatal and adult periods [6, 7]. On the other hand, the hypothalamic and behavioural responses to psychological stress are vulnerable in females to early life dietary influence, while remaining unaffected in males. Thus, females that are overfed in the first three weeks of their lives have increased open arm exploration as adults in the elevated plus maze test for anxiety, indicative of reduced anxiety-like behaviour, but have exacerbated paraventricular nucleus of the hypothalamus (PVN) responses to acute restraint stress; effects that are not seen in males [3].

Understanding the impact of neonatal obesity in female animal models is important given the psychosocial consequences of childhood obesity can significantly differ between the sexes in humans. Girls and women are more likely to display stress-related eating than boys [8–10], with obesigenic consequences. Overweight girls are also more likely to develop depression, anxiety, and eating disorders than overweight boys [11, 12]. Interestingly, obesity is associated with abnormally high circulating ghrelin in adolescent girls, but not boys [13], suggesting ghrelin may be involved differently between the sexes in the obese phenotype.

Ghrelin is an orexigenic 28 amino acid peptide hormone secreted primarily from the stomach that circulates in two major forms; as acylated and des-acylated ghrelin [14]. In healthy adults ghrelin conveys information on negative energy balance to the brain, stimulating feeding [15, 16]. Recent research, including our own, has demonstrated ghrelin’s important role in coordinating hypothalamic and pituitary functions additional to feeding, including the HPA axis responses to stress [17–21]. Ghrelin is elevated by stress [22–24] and acts directly at the growth hormone secretagogue receptor (GHSR1a), the receptor for acyl ghrelin, in the pituitary gland to stimulate adrenocorticotropic hormone (ACTH) release [25–27]. Under conditions of acute stress, ghrelin-induced ACTH release promotes the secretion of glucocorticoids from the adrenal gland, important for terminating further activation of the HPA axis [18]. We hypothesise here that the exacerbated HPA axis responses to stress in neonatally overfed females might be due, in part, to changes in the way ghrelin mediates ACTH release at the level of the pituitary gland.

This hypothesis is supported by findings that circulating ghrelin and ghrelin signalling, at least at the level of the hypothalamus, are altered in neonatally overfed male neonates and adults. In normal neonates, ghrelin acts to regulate the development of hypothalamic connectivity, in particular curtailing over-development of the neuronal connections between the arcuate nucleus (ARC), lateral hypothalamus (LH) and paraventricular nucleus of the hypothalamus (PVN) that co-ordinate feeding behaviour and metabolism [28, 29]. This particular influence of ghrelin follows a surge in circulating leptin that begins at P4 and peaks at P7-10 in rats [30], triggering the development of hypothalamic connectivity [31]. Ghrelin levels then begin to rise during the second week of life and reach a plateau towards the end of the second week (P14), limiting excessive connectivity between hypothalamic subregions [31]. At least in males, this formative role is influenced by neonatal dietary experiences [32–34]. Thus, neonatally overfed male mice have suppressed levels of circulating acylated ghrelin early in life and this is associated with metabolic dysregulation [33]. Neonatally overfed male rats also have suppressed circulating ghrelin, in this case des-acylated ghrelin, coupled with elevated expression of GHSR1a in the ARC [34]. This early disruption to the ghrelin system with neonatal overfeeding is linked to increased neuronal activation in the ARC and the PVN in response to exogenous acyl, but not des-acyl, ghrelin, suggesting the overfed young male brain is more sensitive to the effects of acylated ghrelin than controls [34]. The effects of neonatal overfeeding on circulating ghrelin, ghrelin signalling to the hypothalamus, and ghrelin’s capacity to stimulate ACTH release from the pituitary in females are currently unknown, however.

In this study we therefore tested if neonatal overfeeding in female rats would cause acute and long-term alterations in ghrelin’s capacity to signal the hypothalamus and pituitary gland. We find that neonatal overfeeding in female rats did not influence circulating ghrelin or hypothalamic ghrelin signalling. It did, however, have lasting effects on pituitary function, leading to diminished responsiveness of the neonatally overfed pituitaries to acyl ghrelin-induced ACTH release. These findings suggest early life obesity may lead to hyperactive HPA axis responses to stress in females at least in part by dysregulating ghrelin signalling at the level at the pituitary.

## Materials and methods

### Animals

In these experiments, we used time-mated pregnant Wistar rats, obtained from the Animal Resources Centre, WA, Australia. On arrival at the RMIT University Animal Facility at day 16 of gestation, they were housed at 22°C, 12 hr light/dark cycle (0700–1900 hr). We provided them with *ad libitum* pelleted standard rat chow and water. All procedures described here were in accordance with the National Health and Medical Research Council Australia Code of Practice for the Care of Experimental Animals and the RMIT University Animal Ethics Committee approval. While pregnancy transport stress is a potential factor in long-term physiology, we do not believe it will have affected one of our experimental groups differently to the others. In designing our experiments, we ensure that control and experimental animals are included in all shipments of rats. We assume *a priori* that any litter born that contains fewer than seven live pups is compromised and we do not use this litter or dam in our experiments. Our design of fostering all the pups on the day of birth counterbalances for the possibility that some dams are more stressed during pregnancy than others. Further, pregnancy transport stress while contributing to a number of outcomes, would be expected to elevate glucocorticoids and program exacerbated HPA axis responses to stress [35, 36]. It is noteworthy then that HPA axis responses to psychological stress are not affected in neonatally overfed male rats. We are thus confident that transport stress is unlikely to have affected only one of our groups [3]. Similarly, fostering can have significant long-term effects [37]. However, we deliberately choose to foster all our pups to control for *in utero* environment, including things like pregnancy transport stress affecting dams differently and number of pups conceived. All pups are fostered, no dam receiving any of her biological pups and it is thus unlikely that the neonatally overfed and control groups are differentially affected by any stress of fostering.

### Litter size manipulation

As we and others have previously described [3, 4, 38–40], on postnatal day (P) 0 (the day of birth) we removed all the rat pups from their dams and randomly fostered them to new dams in litters of 12 (control litter; CL) or 4 (small litter, SL, neonatal overfeeding). No dams received any of their own pups. Each new litter was made up of 50% males and 50% females. Sex was determined by a larger genital papilla and longer anogenital distance in male pups than in female pups [41]. Excess pups were culled. This manipulation leads to SL pups being significantly heavier by approximately P7 and heavier throughout life than CL [3, 4, 38].

We used the pups in experimentation at either P7, P12, or P14 or allowed them to grow into adulthood; approximately P70. For those assigned to adult experiments, we separated the pups into same-sex littermate pairs when weaned at P21 and left them undisturbed, expect for the usual animal husbandry. We used only females in the experiments described here. Data from the males of the same litters have been used for other publications [34]. We derived all experimental groups from three or more litters, using a maximum of two pups from the same litter for an experimental treatment.

### Effects of neonatal overfeeding on circulating ghrelin, corticosterone and ACTH

On P7, P14, or approximately P70 we deeply anesthetized a cohort of the rats with Lethabarb (150 mg/kg pentobarbitone sodium, i.p.), decapitated them and collected trunk blood for later assessment of serum ghrelin, as well as plasma corticosterone and ACTH. On P7 and P14, pups remained with the dam until cull and were not fasted. On P70, we removed the food from the rats at lights-on on the day of cull, ensuring each rat was fasted for at least 2 hr prior to cull to standardise satiety levels without inducing negative energy balance. For ghrelin, blood was treated with Pefabloc (1 mg/mL final concentration) in a tube that contained no anticoagulant. Blood was left to clot at room temperature for 30 min then centrifuged at 2500 g for 15 min at 4 ± 2°C. Serum (top layer) was transferred into a fresh tube then 0.5 M HCl was added (final concentration 0.05 M HCl). The samples were mixed, aliquoted and stored at -20°C avoiding freeze-thaw cycles until use.

To determine ghrelin concentrations in our samples, we performed standard commercial ghrelin ELISAs following the manufacturer’s instructions (total ghrelin and “active” (acylated) ghrelin: Millipore, Billerica, MA, USA). Intra-assay variability for the total and acylated ghrelin ELISAs were 0.3–7% and 0.7–1.3% CV, inter-assay variability 1–10% and 1.8–4.5% CV, and lower limits of detection 0.8 pg/mL and 0.04 ng/mL. Samples were assayed in duplicate and all compared samples were processed in the same assay. Acyl ghrelin concentrations were subtracted from total ghrelin concentrations to derive a value for serum des-acyl ghrelin [42].

For the assessment of corticosterone and ACTH we collected blood into EDTA-coated tubes, centrifuged at 1000 g for 15 min at 4°C. We then collected plasma and stored the samples at 20°C until assayed. We used standard corticosterone and mouse/rat ACTH ELISA kits (Abnova Corp., Taipei, Taiwan). ACTH was assessed in samples obtained from adult animals only due to high volume sample requirement for this assay. The inter-assay variability for the corticosterone and ACTH assays were, respectively, 10.6% and 11.5% coefficient of variation (CV), intra-assay variability 5.3% and 7.6% CV, and lower limit of detection 0.28 ng/mL and 0.05 pg/mL.

### Effects of neonatal overfeeding on hypothalamic and pituitary gene expression

To assess whether neonatal overfeeding alters hypothalamic or pituitary expression of the ghrelin receptor *Ghsr*, or the expression of *Mboat4* (ghrelin O-acyl transferase, GOAT, the acylating enzyme), we collected brains and pituitaries from the animals described above and conducted quantitative real-time PCR (qRT-PCR). *Mboat4* expression in the hypothalamus and pituitary is modified in response to changes in the metabolic status *in vivo* and in response to direct stimulation with acyl ghrelin *in vitro* [43]. This evidence suggests that hypothalamic and pituitary *Mboat4* expression may be regulated by different states of energy balance, and potentially modulated by neonatal overfeeding as we have previously seen in males [34]. We also assessed whether neonatal overfeeding affects gene expression of pro-opiomelanocortin (POMC), the ACTH precursor [44], and growth hormone (GH), release of which is robustly stimulated by ghrelin [45], in adult pituitary. We collected whole pituitaries and dissected the hypothalamus. These samples were immediately snap-frozen in liquid nitrogen and stored at -20°C until use. We isolated total RNA using QIAzol reagents and RNeasy Mini Kits (QIAGEN, Valencia, CA, USA). We determined RNA concentrations using a spectrophotometer, NanoDrop 2000/2000c (Thermo Fisher Scientific, Wilmington, DE USA) and 1 μg RNA was transcribed to cDNA using iScript cDNA synthesis kits (Bio-Rad Laboratories, Hercules, CA, USA), according to the manufacturer’s instructions.

We performed qRT-PCR using TaqMan Gene Expression Assays (Applied Biosystems, Mulgrave, VIC, Australia) on a Rotor-Gene Q instrument (Qiagen GmbH, Hilden, Germany). We compared a relative quantitative measure of the target gene expression with an endogenous control, *18s*. See Table 1 for primer details. We analysed mRNA expression using the equation 2^-ΔΔ*C(t)*^, where *C(t)* is the threshold cycle at which fluorescence is first detected significantly above background [46], as described in our previous publications [5, 7, 34, 47, 48]. Data are presented as a fold increase relative to P7 CLs (neonates) or adult CLs (adults).

**Table 1:** Primer details for qRT-PCR.

Target Gene	NCBI Reference Sequence	TaqMan Assay ID	Product Size
*Gh*	NM_001034848.2	Rn01495894_g1	60
*Ghsr*	NM_032075.3	Rn00821417_m1	61
*Mboat4*	NM_001107317.2	Rn02079102_s1	93
*Pomc*	NM_139326.2	Rn00595020_m1	92
*18s*	X03205.1	4319413E	187

### Neuronal activation in response to ghrelin

To assess if neonatal overfeeding was likely to influence the ability of the neonatal hypothalamus to respond to circulating ghrelin, we examined neuronal activation in response to 1 mg/kg s.c. acylated ghrelin, 1 mg/kg s.c. des-acylated ghrelin, or equivalent volume of 0.9% saline at P12. For immunohistochemical analysis of c-Fos as a marker of ghrelin-induced neuronal activation we deeply anaesthetised the rats with Lethabarb 2 hr after injection and perfused them transcardially with phosphate buffered saline (PBS: 4°C, pH 7.4), followed by 4% paraformaldehyde in PBS (PBS: 4°C, pH 7.4). We selected the 2 hr time point for the immunohistochemical assessment of ghrelin-induced c-Fos expression based on our [18, 34] and others’ [49–52] previous work indicating that c-Fos is maximally expressed 2 hr after neuronal activation [53]. We have specifically shown that this time point is valid for the assessment of ghrelin-induced hypothalamic c-Fos immunoreactivity in both neonates and adults [34]. Brains were removed and post-fixed for 4 hr in the same fixative before placing them in 20% sucrose in PBS (4°C). We then cut the neonatal forebrains into 40 μm coronal sections using a cryostat. We cut sections into a one in five series and stored them in sodium azide at 4°C until use. We conducted all experiments between 0900 and 1300 hr to limit the potential effects of circadian rhythms on any parameters measured.

### Immunohistochemistry

Sections through the hypothalamus were immunolabelled for c-Fos. Randomly selected sections from each treatment group were processed at the same time in batches. We incubated a single one in five series of sections from each animal in primary antibody (overnight; 4°C; (c-Fos: 1:10 000; rabbit; Santa Cruz Biotechnology, Santa Cruz, CA, USA), followed by secondary antibody (1.5 hr; 1:500; biotinylated anti-rabbit; Vector Laboratories, Burlingame, CA, USA) and avidin-biotin horseradish peroxidase (HRP) complex (ABC; 45 min; Vector Elite kit; Vector). We then incubated the tissue in diaminobenzidine (DAB) intensified with nickel to visualize the HRP activity. We stopped the reaction when there was optimal contrast between specific cellular and non-specific background labelling. We air-dried the brain sections, dehydrated them in a series of alcohols, cleared them in histolene and coverslipped.

Hypothalamic sections were assessed by an experimenter blinded to treatment groups. We counted c-Fos-positive cells in four sections 120 μm apart between 2.52 and 3.12 (PVN) and 2.76 and 3.48 (ARC) mm caudal to bregma for each animal. We saw no differences between the rostrocaudal levels for any of the regions, so we took the sum of the c-Fos-positive cells across all four sections for each animal as our sampled result for that animal.

### Effects of neonatal overfeeding on ghrelin and CRH-stimulated pituitary function *in vitro* in adulthood

To assess the *in vitro* effect of acylated ghrelin on the anterior pituitary release of ACTH and GH, we deeply anaesthetised CL and SL adult rats with Lethabarb, excised their anterior pituitaries and stored the pituitaries in ice-cold Dulbecco’s modified Eagle’s medium/Nutrient mixture F-12 (DMEM/F-12; Thermo Fisher Scientific, Scoresby, Victoria, Australia) containing 0.1% BSA until all tissues were collected. We then bisected each anterior pituitary, weighed and pre-incubated each half pituitary for two x 1 hr in 1 mL of DMEM/F-12 at 37°C in a 95% O_2_/5% CO_2_ atmosphere. After the pre-incubation period, we refreshed the medium and collected samples every 15 min up to 1 hr to obtain basal GH and ACTH release profiles. To assess the pituitary responsiveness to secretagogue stimuli, acyl ghrelin (10^−6^ M) and corticotropic-releasing hormone (CRH; 100 ng/mL)-containing media were added in the second fraction. At the end of each 15 min period the medium was collected and stored in -20°C until assayed. The protocol was adapted from Cai et al. [47]. ACTH and GH levels were assessed by ELISAs following the manufacturer’s instructions. Intra-assay variability for the ACTH ELISA (MD Biosciences, St. Paul, MN, USA) was 3.1%-4.2% CV, inter-assay variability, 5.8%-6.2% CV, and lowest limit of detection, 0.46 pg/mL. Intra-assay variability for the GH ELISA (Millipore, Billerica, MA, USA) was 1.7–4.3% CV, inter-assay variability, 3.2–4.9% CV, and lowest limit of detection, 0.07 ng/mL. Data are expressed as percentage of the basal ACTH and GH secretion as measured at the end of the first 15 min period, and set to 100%.

### Data analysis

We analysed neonatal and adult changes separately. For neonates we compared ghrelin, gene expression, and hypothalamic responses to ghrelin using multi-factorial analysis of variance (ANOVA)s with litter size (CL/SL) and age (P7/14) as between factors. Weights were analysed using a Student’s unpaired t-test for P12, since we have previously reported P7 and P14 weights for females [5]. We also included treatment factors where appropriate (saline/ghrelin). Where significant interactions were found, we then performed Tukey *post hoc* tests. For the adults we used Student’s unpaired t-tests or two-way ANOVAs. ACTH and GH concentrations were compared using repeated measures ANOVAs with litter size and treatment as the between factors and time as the repeated measure. A Bonferroni correction was applied to adjust for multiple comparisons. When the assumption of sphericity was violated, we used the Greenhouse-Geisser correction. Where significant interactions were found, we performed Student’s unpaired t-tests for each timepoint. Area under the curve (AUC) analyses were conducted to assess variations in ACTH and GH levels in the *in vitro* experiments, using (*t*1 + *t*2)/2 + (*t*2 + *t*3)/2 + (*t*3 + *t*4)/2, where *t*n is the hormone value at the n timepoint. Data are presented as the mean + SEM. Statistical significance was assumed when *p* ≤ 0.05.

## Results

### Neonatal overfeeding has minimal effects on the neonatal hypothalamic ghrelin system in females

**Fig 1:**
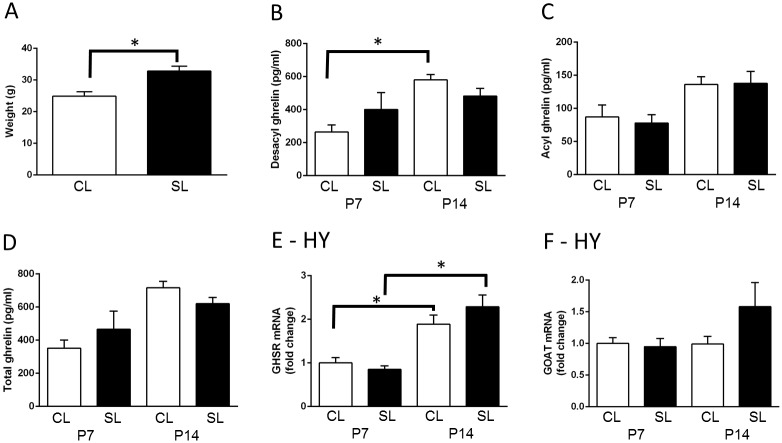
Effects of neonatal overfeeding on the neonatal ghrelin system. A) neonatal body weight, postnatal day (P) 12; n = 6 CL, 5 SL. B-D) serum ghrelin concentrations in control (CL) and neonatally overfed (SL) rats at P7 and P14; n = 8 for all groups. E) hypothalamic growth hormone secretagogue receptor (*Ghsr*) mRNA expression and F) ghrelin O-acyl transferase (GOAT; *Mboat4*); n = 8 CL P7, SL P7, CL P14, 7 SL P14. Data are mean + SEM. * *p* < 0.05. There was a significant main effect of age on acyl and total ghrelin, but no *post hoc* group differences in this measure. *Post hoc* differences between the groups are indicated by joining lines and *.

As we have previously demonstrated [3–5, 7, 38], neonatal overfeeding led to significant early weight gain so that the neonatally overfed females were significantly heavier than controls at P12 (t_(9)_ = 3.89, *p* = 0.004; n = 5–6; Fig 1A). Des-acyl ghrelin levels were differentially affected by age in female rats (significant litter size by age interaction: F_(1,26)_ = 4.48, *p* = 0.044), so that there was a significant small increase in des-acyl levels at P14 in CL, but not SL females. There was also a significant effect of age on acyl (F_(1,26)_ = 14.91, *p* = 0.001) and total ghrelin (F_(1,26)_ = 19.79, *p* < 0.001; Fig 1B–1D). Hypothalamic *Ghsr* and *Mboat4* were not affected by neonatal overfeeding (Fig 1E and 1F) but there was a significant effect of age on *Ghsr* with both groups having more *Ghsr* at P14 than P7 (F_(1,27)_ = 42.82, *p* < 0.001; n = 7–8 per group).

**Fig 2:**
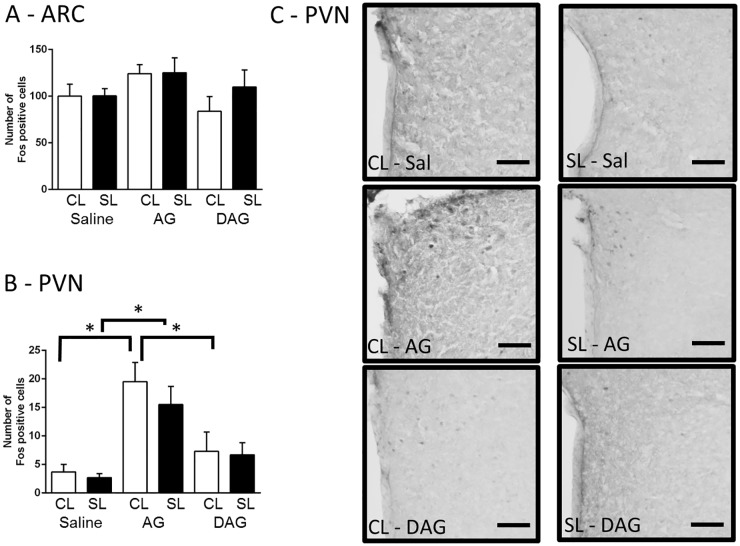
Effects of neonatal overfeeding on the neonatal response to ghrelin. A) Neuronal activation in response to an i.p. ghrelin injection in the arcuate nucleus (ARC) (n = 6 CL Saline, SL Saline, CL AG, CL DAG, 5 SL AG, 7 SL DAG) and B) paraventricular nucleus of the hypothalamus (PVN) (n = 6 CL Saline, SL Saline, CL AG, SL AG, CL DAG, 7 SL DAG). C) Photomicrographs of the PVN. Scale bar = 100 μm. Data are mean + SEM. * *p* < 0.05. *Post hoc* differences between the groups are indicated by joining lines and *.

To test if neonatal overfeeding alters the ability of neonates to respond to a ghrelin signal at this age, we gave them a single injection of acylated ghrelin, des-acylated ghrelin, or saline at P12 and examined hypothalamic c-Fos expression. There was no difference in neuronal activation after acylated or des-acylated ghrelin in the ARC (Fig 2A), despite this dose having a significant effect in males [34]. We did see a significant increase in c-Fos in the PVN in the rats that were given acylated ghrelin (significant effect of drug: F_(2,31)_ = 16.45, *p* < 0.001; n = 5–6; Fig 2B and 2C), but there was no effect of neonatal overfeeding on this response.

### Neonatal overfeeding does not affect the adult hypothalamic ghrelin system in females

**Fig 3:**
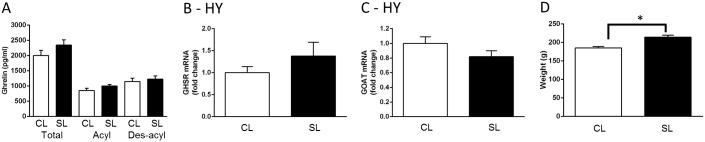
Effects of neonatal overfeeding on the ghrelin system long-term. A) adult serum ghrelin concentrations. B) hypothalamic growth hormone secretagogue receptor (*Ghsr*) and C) ghrelin O-acyl transferase (GOAT; *Mboat4*) mRNA expression; n = 6 CL, 8 SL for A-C. D) adult body weight n = 6 for all groups. Data are mean + SEM. * *p* < 0.05. T-test differences between the groups are indicated by joining lines and *.

To assess the long-term effects of neonatal overfeeding on the ghrelin system, we examined circulating ghrelin and hypothalamic expression of *Ghsr* and *Mboat4* (GOAT) mRNA of adult females that had been overfed as neonates. There was no effect of neonatal overfeeding on serum ghrelin levels in these females (Fig 3A). There was also no effect on hypothalamic *Ghsr* or *Mboat4* expression (Fig 3B and 3C), despite a persistent increase in body weight in neonatally overfed females (t_(10)_ = 4.36, *p* = 0.001; n = 6; Fig 3D), indicating neonatal overfeeding leads to persistent weight gain without disrupting the hypothalamic ghrelin system in female rats.

### Neonatal overfeeding leads to lasting changes in pituitary ghrelin signalling in adult females

We next assessed whether neonatal overfeeding might affect the potential for ghrelin to influence stress responses by disrupting its ability to modulate ACTH release at the level of the pituitary, either acutely or long-term.

#### Pituitary *Ghsr* and *Mboat4* gene expression

**Fig 4:**
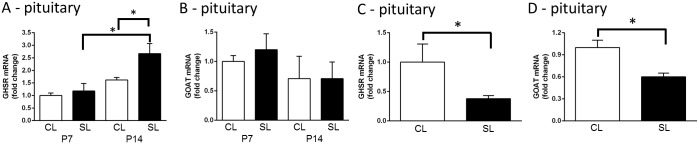
Effects of neonatal overfeeding on the ghrelin pituitary signalling long-term. A) Neonatal pituitary growth hormone secretagogue receptor (*Ghsr*); n = 8 CL P7, CL P14, 7 SL P7, 6 SL P14, and B) pituitary ghrelin O-acyl transferase (GOAT; *Mboat4*) mRNA expression; n = 7 CL P7, SL P7, CL P14, 6 SL P14. C) Adult pituitary *Ghsr* and D) pituitary *Mboat4* mRNA expression in control (CL) and neonatally overfed (SL) rats; n = 6 CL, 8 SL. Data are mean + SEM. * *p* < 0.05. *Post hoc* and t-test differences between the groups are indicated by joining lines and *.

There was a significant increase in *Ghsr* mRNA in the pituitaries of neonatally overfed compared with control rats at P14 (significant effect of litter size: F_(1,25)_ = 6.89, *p* = 0.015 and age: F_(1,25)_ = 20.46, *p* < 0.001; n = 6–8; Fig 4A), indicating that pituitary’s capacity to respond to ghrelin may be altered at this age. There was no effect on *Mboat4* expression in the same animals (Fig 4B). There was also no effect on basal corticosterone levels to suggest unstimulated HPA axis was affected: CL P7 = 25.9 ± 2.2, n = 5; SL P7 = 29.7 ± 3.5, n = 6; CL P14 = 34.6 ± 7.8, n = 5; SL P14 = 38.6 ± 7.0 ng/mL, n = 6.

There were notable persistent effects of neonatal overfeeding on pituitary *Ghsr* and *Mboat4* expression long-term, interestingly in the opposite direction to the neonatal effect. Thus, neonatal overfeeding significantly suppressed both pituitary *Ghsr* (t_(12)_ = 2.29, *p* = 0.041; n = 6–8) and *Mboat4* (t_(12)_ = 2.61, *p* = 0.023; n = 6–8) in the adult females compared with controls (Fig 4C and 4D). In the adults, basal circulating corticosterone (CL = 55.2 ± 13.7, SL = 41.7 ± 20.0 ng/mL; n = 7 / group) and ACTH (CL = 44.4 ± 7.5, SL = 29.2 ± 4.7 pg/mL; n = 9 CL, 5 SL) were also not affected.

#### Unstimulated and stimulated *in vitro* pituitary GH release

**Fig 5:**
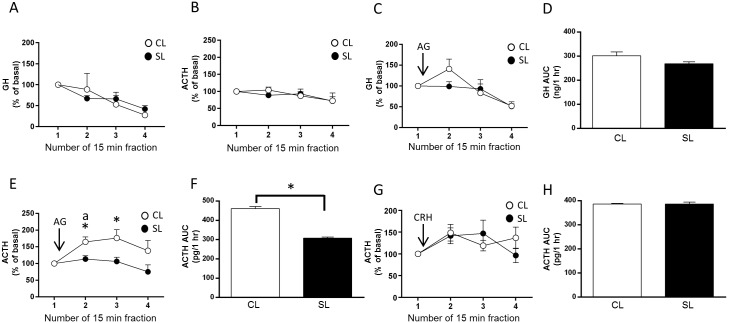
Effects of neonatal overfeeding on the adult pituitary responsiveness to ghrelin and Corticotropic-Releasing Hormone (CRH). A) Basal release of growth hormone (GH) in pituitaries from adult neonatally overfed (SL) and control (CL) rats; n = 4 for all groups. B) Basal release of adrenocorticotropic hormone (ACTH); n = 5 CL, 4 SL. C) Acyl ghrelin-induced GH release in pituitaries from adult SL and CL rats; n = 5 CL, 6 SL. Acyl ghrelin (10^−6^ M) was added after collection of fraction 1. D) Area under the curve (AUC) analysis of acyl ghrelin-stimulated GH release. E) Acyl ghrelin-induced ACTH release; n = 11 CL, 8 SL. Acyl ghrelin (10^−6^ M) was added after collection of fraction 1. F) AUC analysis of acyl ghrelin-induced ACTH release. G) Corticotropic-releasing hormone (CRH) -induced ACTH release; n = 4 CL, 5 SL. CRH (100 ng/ml) was added after collection of fraction 1. H) AUC analysis of CRH-induced ACTH release. Data are mean + SEM. * *p* < 0.05. C: significant main effect of fraction, E: significant main effect of fraction and litter size with *post hoc* differences between litters indicated by * and *post hoc* differences from fraction one indicated by ‘a’. G: significant main effect of fraction.

We next considered whether this suppression of *Ghsr* expression in the neonatally overfed adult pituitary would affect the pituitary’s ability to respond to upstream signals. There was a significant effect of time on the levels of GH in the unstimulated media, with no *post hoc* differences (within-subjects significant effect of time: F_(3,18)_ = 6.51, *p* = 0.004; n = 4; Fig 5A) and no effect of time on ACTH (Fig 5B). We found no effect of neonatal overfeeding on the basal *in vitro* release of GH or ACTH over time (Fig 5A and 5B). GH secretion from the pituitary is directly and robustly stimulated by ghrelin [45]. In our neonatally overfed and control rats there was a significant effect of time on acyl ghrelin-induced GH levels, with no further *post hoc* differences (within-subjects significant effect of time: F_(3,27)_ = 12.01, *p* = 0.001; n = 5–6; Fig 5C) and no differences between the groups with the AUC analysis (Fig 5D), indicating the GH response to acyl ghrelin is intact in the neonatally overfed females.

#### Unstimulated and stimulated *in vitro* pituitary ACTH release

Neonatal overfeeding did, however, affect pituitary ACTH secretion. When stimulated *in vitro* with acyl ghrelin, the control pituitaries responded with a robust increase in ACTH at 15 min that was still elevated with respect to the neonatally overfed at 30 min after acyl ghrelin (within-subjects significant effect of time: F_(3,51)_ = 3.38, *p* = 0.048; between-subjects significant effect of litter size: F_(1,17)_ = 5.57, *p* = 0.031; n = 8–11; Fig 5E). The AUC analysis revealed a significant suppression of the overall production of ACTH in the neonatally overfed pituitaries in response to acyl ghrelin compared with controls (t_(4)_ = 4.03, *p* = 0.016; Fig 5F).

**Fig 6:**
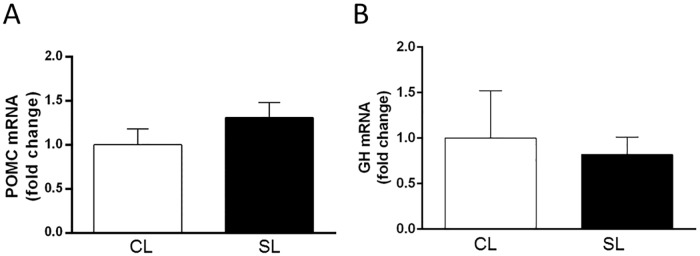
Effects of neonatal overfeeding on pro-opiomelanocortin (*Pomc*) and Growth hormone (*Gh*) mRNA expression in the adult pituitary. A) Adult pituitary pro-opiomelanocortin (*Pomc*); n = 8 CL, 6 SL, and B) growth hormone (*Gh*) mRNA expression; n = 6 CL, 7 SL. Data are mean + SEM.

We next examined whether neonatal overfeeding similarly suppresses ACTH production in response to stimulation with CRH, the main stimulator of ACTH secretion. Stimulation with CRH increased ACTH production as compared to baseline levels and as previously shown in studies using similar protocols to assess CRH-stimulated ACTH production *in vitro* [54, 55]. There were no further *post hoc* differences between fractions or experimental groups (within-subjects significant effect of time: F_(3,21)_ = 2.96, *p* = 0.056; n = 4–5; Fig 5G), and no differences were revealed in the AUC analysis (Fig 5H). Thus, neonatal overfeeding leads to an impairment in ghrelin’s ability to stimulate pituitary ACTH, without affecting the ACTH response to stimulation with CRH. There were no differences in pituitary *Pomc* or *Gh* mRNA to suggest gene expression was affected by neonatal overfeeding (Fig 6A and 6B).

## Discussion

Here we showed that in females neonatal overfeeding did not affect circulating ghrelin, in contrast to our previously observed findings in males [34], nor did it influence the hypothalamic ghrelin system or the hypothalamic ability to respond to exogenous ghrelin, unlike in males [34]. Neonatal overfeeding did, however, alter the pituitary gene expression of GHSR and GOAT in female rats, with an increase in GHSR expression at two weeks of age, followed by a decrease in GHSR and GOAT expression in adult pituitary relative to controls. While the basal GH and ACTH levels, as well as the GH response to ghrelin and ACTH response to CRH remained intact in the neonatally overfed female pituitary, ACTH production was markedly less responsive to ghrelin.

We should note that factors in addition to differences in pre-weaning nutrition are inherent in this model. The model is certainly effective at increasing food consumption by those from small litters. Thus, earlier work shows rat pups raised in small litters receive more milk than those from control litters, despite the dam reducing her milk production to compensate [56]. These pups also have a pronounced increase in their growth trajectory that reflects this extra milk consumption, including earlier eye opening [38]. However, the model has factors additional to the nutritional elements that are likely to contribute to the long-term phenotype. For instance, the neonatally overfed rats may also experience increased maternal attention and care. There appear to be differences in the impact of litter size on maternal care in rats between different studies, with some studies indicating a significant negative correlation between litter size and the quality of maternal care (licking, grooming or arched-back nursing) [57, 58], while others report no such relationship [59, 60]. While we cannot rule out that maternal attention contributes to some of the effects we see in our studies, we should note the outcome of high levels of maternal care in the rat is usually increased efficiency of the HPA axis due to histone acylation-mediated enhanced glucocorticoid receptor transcription [61]. If anything, being suckled in a small litter reduces HPA axis efficiency in female rats (and does not affect it in males [3]), i.e. the opposite effect. Differences in body temperature regulation within the nest and differences in specific nutritive contents of the milk, such as ghrelin, might also contribute to long-term phenotypic differences.

Ghrelin is traditionally recognised for its regulatory role in energy homeostasis [62, 63]. Circulating and peripherally-injected ghrelin activates GHSR-expressing NPY and AgRP neurons in the ARC. These neurons project to the PVN stimulating feeding behaviour (reviewed in [15]). At high doses peripherally administered ghrelin induces c-Fos activation in the PVN [49], although GHSR-containing neurons are negligibly expressed in this region [64, 65]. While direct infusion of acyl ghrelin into the PVN stimulates food intake [66], peripheral ghrelin-induced feeding effects require intact ARC, since ARC-ablated mice do not respond to the orexigenic effects of peripherally administered ghrelin but increase food intake in response to centrally administered ghrelin [49]. Ghrelin also influences HPA axis activity and although GHSR-expressing cells do not co-localise with CRH-producing neurons in the PVN, centrally administered ghrelin increases CRH mRNA [67] and indirectly activates CRH neurons in this region [64]. Previous studies have shown these hypothalamic effects of ghrelin can be altered by varying environmental challenges including early life diet, at least in males. Thus, neonatal overfeeding in males attenuates peripheral ghrelin levels and alters hypothalamic responsiveness to exogenous ghrelin [32, 34]. Our data in the present study indicated neonatal overfeeding in females did not affect the circulating and central ghrelin system as it does in males [32, 34], suggesting sex differences may exist in the way the early life nutritional environment influences ghrelin’s metabolic effects. Several other studies have observed sex differences in circulating ghrelin levels, with increases in basal total ghrelin levels in women [68, 69], as well as exercise- and fasting-induced increases in acyl ghrelin levels in women [70] and total ghrelin levels in women [71, 72] and female rats [73], compared to their male counterparts. Evidence from animal and human studies also indicates increased prevalence of stress-related disorders in females of reproductive age, relative to males [74–78]. Similarly, we have previously demonstrated increases in the stress responsiveness of female neonatally overfed rats, an effect that is not seen in males [3], leading us to investigate the stress-related effects of ghrelin in the context of early life overfeeding.

It is important to note that we did not monitor the estrous cycle in this cohort of animals due to the significant potential for these measurements to cause additional stress. We consider that if cycle-related variability was a substantial contributing factor to our results we would expect to see greater variability in the various measures in females compared to what we have previously seen in males [34], which is not the case. In support of our own observation, a recent meta-analysis of 311 articles in the field of neuroscience reveals that even when female rats are used in neuroscience experiments without controlling for their estrous cycle stage, there is no greater data variability in females than there is in males [79]. In addition, we have previously investigated the impact of neonatal overfeeding on the onset of reproductive maturity and while the onset of estrous cycle occurs earlier in the neonatally overfed [5], there are no differences in the regularity of estrous cyclicity (unpublished observations), suggesting there are no pre-existing differences in the cycle regularity between the groups that could differentially affect one group over the other.

Ghrelin is gaining increasing attention as a mediator of stress, reward, and mood disorders [18, 23, 80–82]. Ghrelin, particularly acyl ghrelin, is acutely elevated by an exposure to stress [22–24], or even in anticipation of stress [82]. A stress-induced increase in acyl ghrelin directly corresponds to the extent of stress, so that men and women with greater glucocorticoid responses to stress also have higher ghrelin levels [22], and women who experience high levels of interpersonal stress have higher levels of total ghrelin than those women who experience fewer interpersonal stressors [83]. We have also previously demonstrated that under conditions of acute stress, acyl ghrelin acts as an anxiolytic factor by acting at the level of the anterior pituitary [18].

In response to stress, CRH is released from the PVN and acts at the anterior pituitary to stimulate the release of ACTH. ACTH then induces the release of glucocorticoids from the adrenal cortex. These then feed back onto the glucocorticoid receptors in the hypothalamus and the hippocampus to suppress further activation [84, 85]. In mice lacking endogenous ghrelin (ghr-/-), activation of the PVN in response to acute stress is exacerbated, but ACTH and glucocorticoid release are reduced compared with wild-type mice. Exogenous glucocorticoids induce similar activation of the PVN, and exogenous ACTH stimulates similar glucocorticoid release in both groups, suggesting that the hypothalamic and adrenal ability to respond to stress is normal in the absence of ghrelin [18]. Since GHSR expression is extensive in the corticotropic cells in the anterior pituitary [18, 86], it is likely that reduced activation of pituitary GHSR in ghr-/- mice is responsible for reduced ACTH levels, diminished negative feedback on the HPA axis and increased anxiety in these animals [18]. Similarly, GHSR null mice demonstrate increased stress-induced depressive behaviours as compared to wild type controls [87], suggesting intact GHSR signalling is necessary to minimise the detrimental effects of stress.

Together, these findings suggest the decreased pituitary gene expression of GHSR we saw in neonatally overfed adult females in the present study may account for the less effective pituitary response to ghrelin in terms of ACTH secretion. Pituitary GOAT expression, that was also decreased in the neonatally overfed females, has been previously shown to be down-regulated in obese animals [43]. Stimulation of mouse corticotroph cells with acyl ghrelin increases GOAT mRNA expression by these cells, indicating that acyl ghrelin may regulate its own production and function at the level of the pituitary [43]. Therefore, reduced gene expression of pituitary GHSR and GOAT in the neonatally overfed adult females conceivably limited acyl ghrelin’s effects on ACTH release and would potentially lead to an impaired ability of the neonatally overfed females to respond to stress *in vivo*. As such, GOAT deletion has been recently shown to result in an anxiogenic phenotype under both stressed and non-stressed conditions, due to reduced negative glucocorticoid feedback in the PVN [88].

Unlike in previous studies [89], we did not see differences in basal corticosterone in the neonates or in corticosterone or ACTH in the adults to indicate the HPA axis is altered under basal conditions, although it would be interesting to test this with different levels of energy balance. It is interesting, however, that *in vivo* both neonatally overfed and control female rats respond with elevated corticosterone levels to restraint stress compared with males. There is, interestingly, an effect of time on the release of corticosterone in the neonatally overfed compared with controls, with a tendency for a diminished corticosterone response in neonatally overfed females at 30 min, but not 60 min post-stress, relative to control females [3]. The timing of hormonal and behavioural changes after stress is particularly relevant when examining the effects of ghrelin, since stress-induced increases in ghrelin or administration of exogenous ghrelin *in vivo* have been shown to exert both anxiolytic and anxiogenic effects. This disparity is likely to be related to the duration of stress, the levels of ghrelin, the timing of assessment after stress or ghrelin challenge, and food availability (reviewed in [17, 90]). Under food-restricted conditions ghrelin promotes food-seeking and increases locomotion, contributing to anxiety-like behaviour, while when food availability is not restricted ghrelin decreases locomotion (reviewed in [17]). Our previous data indicate that under non-stressed conditions ghrelin produces anxiogenic effects, while it acts as an anxiolytic in response to acute stress [18]. These findings highlight the complexity of ghrelin’s role in the regulation of stress responsiveness *in vivo*, and its investigation in the laboratory setting.

Collectively, our data suggest that, unlike in males [34], neonatal overfeeding in females did not alter the hypothalamic ghrelin system. However, neonatal overfeeding did affect pituitary ghrelin signalling in females. These findings suggest early life obesity potentially alters how females respond to stress throughout life via changes to the way ghrelin modifies the stress response at the level of the pituitary. Further studies are needed to establish these effects *in vivo* and if these findings are applicable across species.
